# Promoting Transplant Access Through Dialysis Facility Performance Metrics: A Double‐Edged Sword

**DOI:** 10.1111/1475-6773.70138

**Published:** 2026-06-16

**Authors:** Adam S. Wilk, Stephen O. Pastan, Rachel E. Patzer

**Affiliations:** ^1^ Division of Transplantation, Department of Surgery Indiana University School of Medicine Indianapolis Indiana USA; ^2^ Center for Health Services Research Regenstrief Institute Indianapolis Indiana USA; ^3^ Division of Nephrology, Department of Medicine Emory University School of Medicine Atlanta Georgia USA; ^4^ Emory Transplant Center Atlanta Georgia USA

The Centers for Medicare and Medicaid Services (CMS) is advancing reforms to payment and performance measurement systems, aiming to promote greater transparency and shared accountability throughout the health care system. Notable recent examples include the new ACCESS Model, which incentivizes technology‐enabled care like remote monitoring and digital coaching for individuals with diabetes, musculoskeletal pain, or other chronic conditions, and the GUIDE Model, which supports coordinated collaborative care for individuals with dementia. The Department of Health and Human Services is using these models and other initiatives, such as Meaningful Measures 2.0, to advance the goal of improving care quality through modernized data systems. These initiatives will launch new data collection efforts, integrate new data fields with existing systems, and foster more real‐time data transmission using application programming interfaces (APIs) and other innovative tools.

As highlighted in the February 2026 issue of Health Services Research by Yang and colleagues [1], these trends are reflected in the quality measurement efforts to support individuals with end‐stage kidney disease (ESKD). In this care setting, multiple payment models—the Kidney Care Choices Models, the End‐stage Renal Disease Treatment Choices Model, and most recently the Increasing Organ Transplant Access Model launched in July 2025—have sought to promote quality of care and access to kidney transplantation using performance measures. Promoting kidney transplantation is especially important as a care improvement goal in ESKD treatment because for most patients, transplantation is the optimal treatment strategy, associated with greater life expectancy, improved quality of life, and lower costs than dialysis, the only life‐saving alternative.

## 1 Measuring Dialysis Facilities' Performance Related to Transplant Care

**FIGURE 1:**
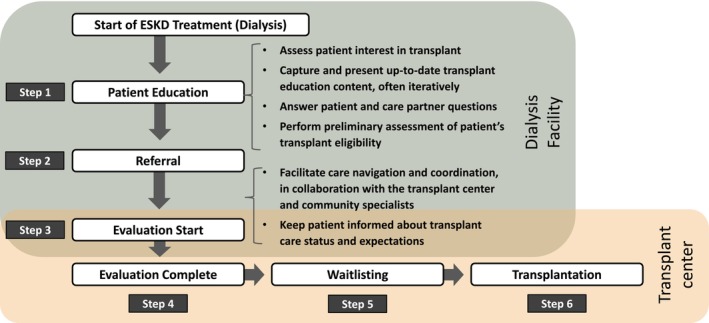
Dialysis facility roles in the kidney transplant care pathway.

Since most transplants happen after patients with ESKD start dialysis treatment, dialysis facilities are well‐positioned to support patients' first steps along the transplant care pathway (Figure 1), and indeed CMS's conditions for coverage mandate that dialysis facilities provide such support. Specifically, dialysis staff are expected to provide patient education and to facilitate informed, shared decision‐making regarding transplantation, leading to the decision to refer the patient for transplant evaluation. Moreover, due to the patient's longitudinal relationship with the dialysis facility, dialysis facility staff can also help coordinate care post‐referral, working with the patient, the transplant center, and other providers to support logistical management (e.g., to promote appointment attendance) and to monitor and share key transplant care information (e.g., the patient's waitlist status) throughout the evaluation process. How well dialysis facilities perform these duties may vary, however; this is reflected in the tremendous variation researchers have observed in transplant referral rates across dialysis facilities [2]. Recent qualitative evidence [3] has tied variation in dialysis facility referral practices to diverging policies, beliefs, and behaviors at the clinician, care team, and facility leadership levels. Consequently, dialysis facility‐level performance measures hold some promise as a tool for standardizing care and, ultimately, improving patients' access to kidney transplantation.

In this context, Yang and colleagues examined CMS's Dialysis Facility Care Compare (DFCC), which serves as the primary venue for publicly reporting dialysis performance measures, presenting this information in star ratings intended to inform patients' dialysis facility choices. Although historically patients have made limited use of such reporting tools [4], enhancing the utility and transparency of this information is essential for driving patient‐centered healthcare decisions. In October 2023, CMS integrated two transplant‐related metrics into the DFCC star rating methodology—the Standardized First Kidney Transplant Waitlist Ratio (SWR) and the Percentage of Prevalent Patients Waitlisted (PPPW). It was anticipated that patients willing to pursue a transplant could use this information to identify which facilities could most effectively help them navigate the transplant care pathway.

While long‐term shifts in provider behavior and patients' transplant access outcomes are of significant interest, Yang et al. [1] utilized 2023 DFCC data (reflecting dialysis facilities' year 2021 performance)—recalculating dialysis facility star ratings twice, both with and without SWR and PPPW included—to measure how often ratings changed and to determine whether certain types of facilities were systematically advantaged or disadvantaged. Given the persistence of inequities in transplant access [5], this study offers insights into the equity implications of these star rating system changes and the potential need for refining risk‐adjustment policies to ensure diverse dialysis facilities are represented fairly in systems that rely on these performance measures.

Yang et al. found that the inclusion of the SWR and PPW measures in DFCC star ratings was associated with a change in star rating (increase or decrease) for 36.5% of dialysis facilities. This frequency is large enough to signal that the newly incorporated measures capture meaningfully distinct information that was previously not captured in star rating measures. The authors also found that star rating increases (vs. decreases) were 5–10 percentage points less likely among facilities in low socioeconomic status communities, in rural communities, those serving above‐median proportions of dual Medicare‐Medicaid‐eligible patients, or those operated by large dialysis organizations, among other characteristics examined. In summary, Yang et al. found that incorporating these new transplant‐focused measures in DFCC star ratings will affect resource‐constrained facilities more negatively.

## 2 Implications for DFCC Star Ratings and Risk Adjustment

Drawing on their findings, Yang et al. offer arguments both in support of and in opposition to the inclusion of these measures and risk adjustment for them. Two key questions should drive how we weigh these arguments. First, *does variation in performance on these measures reflect underlying differences in their patients and communities that could affect patients' progress toward waitlisting*, thus justifying risk adjustment for social risk factors? And second, *does variation in performance on these measures reflect differences in the quality of dialysis facilities' transplant‐related care*, such that patients interested in transplant should be encouraged to use this information and seek care at higher‐rated facilities?

To the first question, studies have documented how low socioeconomic status (e.g., education, health education, insurance, and access to personal transportation) can significantly affect patients' progress through early transplant care steps [6, 7, 8]. Transplant centers typically screen for several of these factors when determining patients' transplant eligibility [8, 9]. Consistent with this, Yang et al. show that SWR and PPW measure performance is significantly influenced by socioeconomic factors. Thus, dialysis facilities in low‐SES communities *are more likely* than their peers to be penalized (i.e., have lower star ratings) as a result of DFCC incorporating these measures. Risk adjustment for relevant social risk factors represents one strategy for avoiding this unbalanced impact.

Making inferences related to the second question is harder. To our knowledge, no national studies have documented variation across dialysis clinics in transplant‐related care practices and policies (e.g., when transplant education is provided, who provides it, which patients are encouraged to pursue transplant, and which are discouraged). Yet it seems likely that many dialysis facilities have insufficient staffing, transplant‐related education, and financial flexibility to take actions to meaningfully improve transplant care quality where any deficits might be observed. Dialysis facilities with fewer resources, where clinicians may already struggle with delivering high‐quality dialysis care [10, 11], may be particularly ill‐equipped to enhance their transplant‐related care. Interventions to facilitate transplant referral in dialysis facilities have been tested: we have facilitated the development of a few interventions that can help in improving transplant access, and we are aware of several others. Yet none can be expected to work in every dialysis care setting, none is adopted widely, and many dialysis facility leaders may not feel equipped to implement any they learn about. Moreover, for many dialysis facilities, helping patients to address deficits in health‐related social needs such as fostering social support, which may be essential for improving some transplant outcomes, is commonly considered beyond the scope of practice for dialysis professionals. Thus, even to the extent SWR and PPW measures reflect some dialysis facilities' practices, it would be challenging for many to improve. In this sense, policy leaders may consider SWR and PPW measures inadequate to promote accountability for transplant care in dialysis care settings.

It remains important to reflect on the utility of star ratings from the patient's perspective as well. Notably, a star rating does not explain in which domains of care a dialysis facility is weak or strong, including in terms of patients' transplant access outcomes. While disaggregated results are also available, as Yang et al. observe, in DFCC the star ratings attract the most attention from patients. For many patients with ambitions to pursue a transplant, the challenge of distinguishing between dialysis facilities with strong (vs. weak) transplant care supports may undermine the utility of DFCC and the intentions behind its new transplant‐related performance measures [4].

In terms of risk adjustment, Yang et al. underscored the double‐edged sword of transplant‐related performance measurement in dialysis facilities: while dialysis facilities have opportunities to exert some meaningful influence over waitlisting outcomes, community‐level social risk factors remain powerful, independent determinants. Therefore, when these performance measures are not appropriately adjusted, patients may misattribute poor outcomes to dialysis facilities when they are driven instead by patient and community factors. This suggests a compelling rationale for maintaining SWR and PPPW metrics within the DFCC, provided they are coupled with robust risk adjustment for factors that predispose specific patient populations to poor outcomes.

Accounting for multidimensional social risk remains a significant methodological challenge. While some factors—including dual eligibility and community‐level indices like the Area Deprivation Index (ADI)—are routinely captured, others are less transparent. Recent additions to the CMS 2728 form regarding housing, transportation, and health literacy represent progress, yet these single items contain only limited information, and information on critical variables like immigration status and transplant‐specific social support is absent. Furthermore, the recent discontinuation of race and ethnicity data collection at the time of ESKD treatment initiation on the CMS 2728 form (among other federal forms) removes a vital proxy for structural racism and broader socioeconomic disadvantage, complicating equitable adjustment [12].

Beyond these data limitations, there remains a broad lack of precedent in policy for non‐clinical risk adjustment, despite emerging (and then fading) examples like the “Health Equity” adjustment in Medicare's ESRD Treatment Choices (ETC) model. Because patients, policy leaders, and health systems' goals for risk adjustment in transplant‐related performance measurement may differ, efforts are needed to assess any misalignment and build consensus to support implementing goal‐aligned risk adjustment models.

## 3 Conclusions

Dialysis facilities play pivotal yet underrecognized roles in supporting patient access to early steps of the transplant care pathway. CMS has aimed to bridge this gap by publicly reporting measures of transplant‐related care quality among dialysis facilities. Yang et al.'s study lays bare that current dialysis facility performance measurement (especially without strong social risk adjustment) risks misrepresenting facility quality with respect to transplant education and policies, which could mislead patients. Continuing partnerships among policymakers, health systems, and researchers are needed to strengthen data infrastructure and clarify policy goals in performance measurement and risk adjustment to ensure alignment with the broader goals of promoting transplant access in dialysis facilities and throughout the transplant care system.

## Funding

Dr. Adam S. Wilk reports funding from NIDDK (K01DK128384).

## Conflicts of Interest

The authors declare no conflicts of interest.

## Data Availability

Data sharing not applicable to this article as no datasets were generated or analyzed during the current study.
